# Skewed distribution of spines is independent of presynaptic transmitter release and synaptic plasticity, and emerges early during adult neurogenesis

**DOI:** 10.1098/rsob.230063

**Published:** 2023-08-02

**Authors:** Nina Rößler, Tassilo Jungenitz, Albrecht Sigler, Alexander Bird, Martin Mittag, Jeong Seop Rhee, Thomas Deller, Hermann Cuntz, Nils Brose, Stephan W. Schwarzacher, Peter Jedlicka

**Affiliations:** ^1^ Faculty of Medicine, ICAR3R—Interdisciplinary Centre for 3Rs in Animal Research, Justus Liebig University Giessen, Rudolf-Buchheim-Straße 6, Giessen, D-35392, Germany; ^2^ Neuroscience Center, Institute of Clinical Neuroanatomy, Goethe University Frankfurt, Frankfurt am Main, 60528, Germany; ^3^ Department of Molecular Neurobiology, Max Planck Institute of Multidisciplinary Sciences, Göttingen, 37077, Germany; ^4^ Frankfurt Institute for Advanced Studies, Frankfurt am Main, 60438, Germany; ^5^ Ernst Strüngmann Institute (ESI) for Neuroscience in Cooperation with Max Planck Society, Frankfurt am Main, 60528, Germany

**Keywords:** lognormal distribution, heterosynaptic plasticity, Munc13 double-knockout mice, multiplicative noise, intrinsic/extrinsic synaptic dynamics, spike-timing-dependent plasticity (STDP)

## Abstract

Dendritic spines are crucial for excitatory synaptic transmission as the size of a spine head correlates with the strength of its synapse. The distribution of spine head sizes follows a lognormal-like distribution with more small spines than large ones. We analysed the impact of synaptic activity and plasticity on the spine size distribution in adult-born hippocampal granule cells from rats with induced homo- and heterosynaptic long-term plasticity *in vivo* and CA1 pyramidal cells from Munc13–1/Munc13–2 knockout mice with completely blocked synaptic transmission. Neither the induction of extrinsic synaptic plasticity nor the blockage of presynaptic activity degrades the lognormal-like distribution but changes its mean, variance and skewness. The skewed distribution develops early in the life of the neuron. Our findings and their computational modelling support the idea that intrinsic synaptic plasticity is sufficient for the generation, while a combination of intrinsic and extrinsic synaptic plasticity maintains lognormal-like distribution of spines.

## Graphical abstract

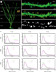

## 1. Introduction

A variety of features in the brain including dendritic spine size [1–3], synaptic strength [4–7] and neuronal firing rate [8] are strongly positively skewed with a heavy tail, displaying a lognormal-like distribution. Lognormal-like distributions of synaptic and firing rate parameters are thought to play a fundamental role in the structural and functional organization of the brain [9–11], and a number of explanations for the emergence of such distributions in active and plastic networks have been proposed.

Spines are plastic and motile structures of neuronal dendrites that function as postsynaptic sites for excitatory inputs. The spine head contains the postsynaptic density (PSD) with AMPA and NMDA glutamate receptors [12]. The size of the PSD correlates with spine head size, the number of presynaptic vesicles [13,14] and the density of postsynaptic receptors [15–18]. Therefore, spine head size has been used as a morphological proxy for synaptic strength [19,20]. Spines change in size, shape and number depending on synaptic activity (for reviews see [21–26]), which has been termed extrinsic spine size dynamics [11].

Given the overwhelming evidence for activity-dependent, extrinsic spine dynamics, the conventional view would be to expect spine size distributions to depend heavily on synaptic activity and associated synaptic plasticity [9] (see also [27–29]). However, spines also display spontaneous, activity-independent, intrinsic changes [30,31]. In keeping with a major role of such intrinsic spine dynamics, recent data from pharmacologically silenced cultured rat cortical neurons challenged the conventional view, indicating that skewed synapse weight distributions can emerge in an activity-independent manner [32]. However, what remains unclear are the important questions as to (i) what kind of spine size distributions emerge during dendritic maturation of adult newborn neurons, when and whether these are affected by homo- and heterosynaptic plasticity and (ii) whether such skewed synapse weight distributions can emerge spontaneously in intact neuronal circuits. To address these issues, we studied the distribution of spine sizes in adult-born dentate granule cells (GCs) from rats with induced *in vivo* homo- and heterosynaptic long-term plasticity. In addition, we studied spine size distribution in Munc13 double-knockout (DKO) mouse brain circuits with completely blocked presynaptic activity. We found that homosynaptic long-term potentiation (LTP), with associated spine growth, and heterosynaptic long-term depression (LTD), with associated spine shrinkage, do not disrupt the lognormal-like spine size distribution but rather modulate its parameters. Moreover, we report that the lognormal-like distribution of spine sizes emerges even with entirely blocked synaptic activity.

## 2. Results

### 2.1. Independence of spine size distribution from long-term homo- and heterosynaptic plasticity in adult-born hippocampal granule cells

**Figure 1.**
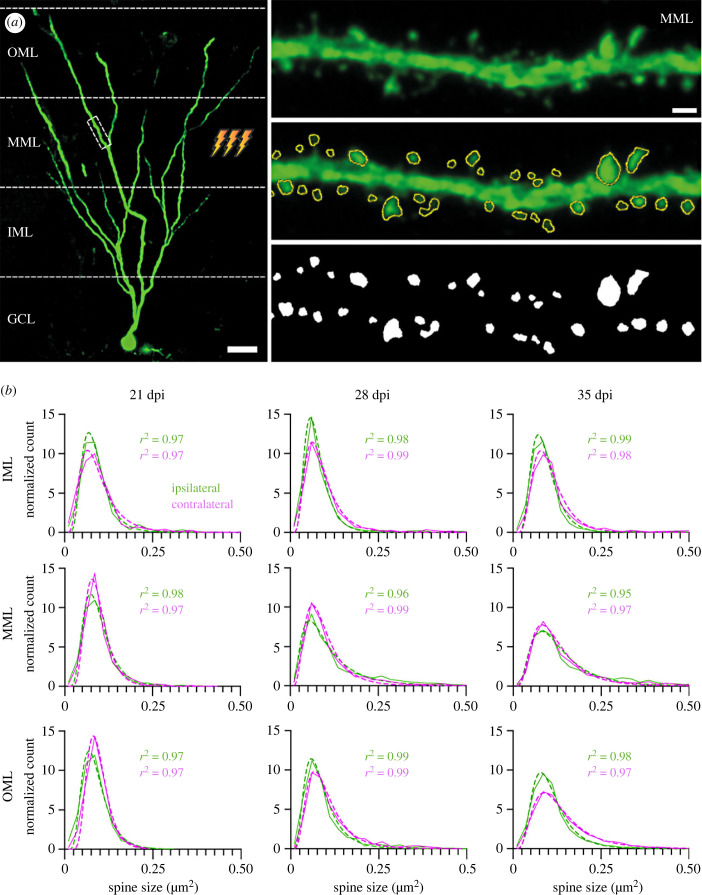
Collected spine size data from anaesthetised rat abGCs reveal robust lognormal-like spine size distributions in all dentate layers and cell ages irrespective of ipsilaterally induced homosynaptic or heterosynaptic plasticity. (*a*) Left: An example retrovirally labelled abGC imaged at 35 days post-injection (dpi; scale bar: 25 µm). The ipsilateral MML experienced 2 h high-frequency stimulation (HFS). Right: Top panel shows an enlarged dendritic segment located in the stimulated ipsilateral MML. Middle, bottom panel depicts analysed spines (scale bar: 1 µm). (*b*) Spine size distributions and their average lognormal fits for all cells in one layer (OML, MML, IML), time (21, 28 and 35 dpi = cell age) and hemisphere (ipsilateral stimulated = green and contralateral control = magenta), fitted to the spine data. Note the high overall goodness of fit for all conditions (mean *r*^2^ over all cells in each condition). The lower ipsilateral versus contralateral (stimulated versus control) distribution peak associated with reduced distribution width in the stimulated MML indicates homosynaptic spine expansion; the higher ipsilateral versus contralateral distribution peak in the OML and IML indicates heterosynaptic spine shrinkage. At 21 dpi, the size distributions show more variance compared to the later time points. This is likely due to spines still maturing and a more random spine growth and shrinkage, whereas at 28 dpi and 35 dpi, spine growth or shrinkage is the result of homo- and heterosynaptic plasticity. This mirrors the findings of Jungenitz *et al*. [33]). OML, MML and IML: outer, middle and inner molecular layers, respectively; GCL: granule cell layer of the DG. The dashed line represents the lognormal fit, the solid line the spine data binned into size categories.

As the effects of nerve cell age and long-term synaptic plasticity on the skewness of spine size distributions are unknown, we characterized the spine size distribution and its relationship to long-term synaptic plasticity in retrovirally labelled adult-born hippocampal granule cells (abGCs) of three different cell ages. These are characterized by gradual onset and development of homo- and heterosynaptic plasticity (21, 28 and 35 dpi; see Methods) [33], soon after start of spinogenesis at 16–18 dpi [34,35]. In these cells, homosynaptic LTP associated with spine enlargement was induced in the middle molecular layer (MML) following 2 h stimulation of the medial perforant path (MPP) [33] *in vivo*. At the same time, concurrent heterosynaptic LTD associated with spine shrinkage was induced in dendrites in the adjacent unstimulated outer and inner molecular layers (OML, IML). Those effects were restricted to the stimulated ipsilateral hemisphere and therefore the unstimulated contralateral site served as control. Here, we fitted a lognormal function to the raw data (21 dpi, *n* = 3 animals; 28 dpi, *n* = 6; and 35 dpi, *n* = 5) to test whether it provides a good fit for the size distribution of mushroom spines. In the first round of analyses, this was done collectively for all of the cells of one condition (i.e. synaptic layer, cell age and hemisphere) together (figure 1).

In all conditions—in ipsi- and contralateral dentate gyrus (DG), at all cell ages and in all three layers—the lognormal-like distribution matched the data exceptionally well with very high goodness of fit (*r*^2^) values of 0.95–0.99 calculated over all cells and animals in each condition. As expected, changes in the shape (peak and width) of the distribution reflected the overall homosynaptic spine enlargement in the ipsilateral MML with respect to the contralateral MML as well as the overall heterosynaptic spine shrinkage in the ipsilateral OML and IML with respect to the contralateral OML and IML. This confirms that after plasticity induction, the number of large spines increased and the number of small spines decreased in the stimulated layer while opposite changes occurred in the adjacent unstimulated layers [33]. However, the lognormal form of the distribution remained.

To see if a skewed, lognormal-like distribution also appeared at the level of individual cells, we examined spines in each cell separately. Both ipsilateral and contralateral (electronic supplementary material, figures S1 and S2) dentate abGCs showed highly rightward skewed distributions at all cell ages and in all layers with a variety in shapes, peaks and widths, and a lognormal-like spine size distribution was observed in all individual cells.

**Figure 2.**
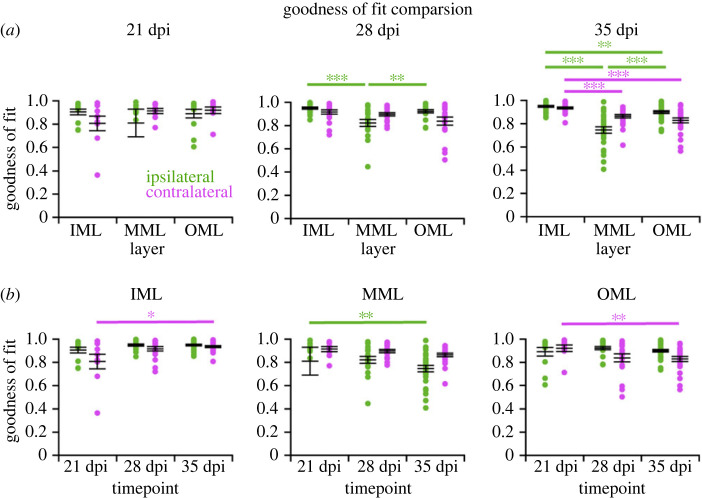
Individual cell level analysis of the spine size data from anaesthetized rat abGCs confirms robust lognormal-like distribution of spine sizes in all dentate layers and cell ages. (*a*) Goodness of fit was similar in the ipsilateral (stimulated; green) and contralateral (control; magenta) DG layers. Significant differences can be observed ipsilaterally; the MML has a significantly lower *r*^2^ compared to the other two layers at 28 and 35 dpi. On the contralateral side, the goodness of fit is significantly lower in both the MML and the OML compared to the IML at 35 dpi. Left, middle, right panel: 21, 28 and 35 dpi, respectively. (*b*) The goodness of fit in each layer changes significantly over time: in the IML, *r*^2^ increases contralaterally from 21 to 35 dpi, whereas in the MML and OML, it decreases ipsi- and contralaterally over the same time period. Left, middle and right panel: IML, MML and OML, respectively. Each point represents a single cell: 21 dpi ipsilateral: *n* = 12 cells, 21 dpi contralateral: *n* = 9; 28 dpi ipsilateral: *n* = 18, 28 dpi contralateral: *n* = 18; 35 dpi ipsilateral: *n* = 30, 35 dpi contralateral: *n* = 24. Error bars represent s.e.m. with mean. The *y*-axes are truncated at 0, with one outlier below this value in the ipsilateral MML at 21 dpi. OML, MML and IML: outer, middle and inner molecular layers, respectively.

To quantify the comparison of spine size distributions between ipsilateral (stimulated) DG with induced synaptic plasticity and the contralateral (control) side, we calculated the cell individual goodness of fit (*r*^2^) (figure 2) and skewness (electronic supplementary material, figure S3). Overall, we achieved a good fit, with the majority of *r*^2^ values for individual cells between 0.8 and 0.99. There was some variability in the goodness of fit as fewer samples (up to 72) were available for analysis compared to more mature cells (up to 105 spines), and one outlier was as low as −0.5 (MML ipsilateral, at 21 dpi). The generally high *r*^2^ values indicate a lognormality of the data at the individual cell level, independent of cell age, cell layer or stimulation (hemisphere). Thus, the rightward skewness of the spine size distribution is a robust and synaptic plasticity-independent phenomenon that is already present at an early neuronal age.

There were no significant differences (*p* < 0.05) in the goodness of fit between the two hemispheres; however, layer and time comparisons show some significant differences (figure 2). The goodness of fit decreased significantly ipsilaterally in the MML compared to the IML and OML at 28 and 35 dpi, suggesting that the plasticity induction weakens the lognormal distribution. This suggests that the stimulation slightly weakens the lognormality of the spines and thus the goodness of fit decreases as well. Contralaterally, there was a significant decrease in goodness of fit in both the MML and OML compared to the IML. Both ipsilateral and contralateral hemispheres showed significant differences from 21 to 35 dpi, in all three layers. This could be an effect of ongoing maturation. Another way to quantify the lognormality of the spine size data is to calculate the skewness (asymmetry around the mean) of the data. All cells in every condition displayed a skewness above 0, confirming that the data were not symmetrically distributed but skewed to the right (electronic supplementary material, figure S3). Again, there were no significant differences between the hemispheres. However, there were significant differences when comparing layers and time points, with skewness increasing over time both ipsi- and contralaterally. Similar to the goodness of fit comparison, this could be a result of cell maturation. At 28 and 35 dpi, the skewness of the stimulated MML is significantly lower compared to both IML and OML, similar to the goodness of fit results above. Overall, the skewness quantification supported the results obtained by the *r*^2^ comparisons, showing that the lognormal-like distribution of spine sizes is independent of stimulation-induced homo- and heterosynaptic plasticity. Comparing the standard deviations taken from the natural logarithms of the spine data (in the following called sigma), which is an indicator of the width of the distribution and in this case the range of the spine sizes, some significant differences (*p* < 0.05) were observed (electronic supplementary material, figure S4). The sigma value for the stimulated ipsilateral MML at 28 dpi significantly increased compared to the contralateral side. This indicates that the shape widened and that there was an increase in bigger spines due to the induction of homosynaptic LTP. There was a significant decrease in the ipsilateral spine sizes in the IML at 21 dpi and the OML at 35 dpi compared to the contralateral side, indicating that the shape narrowed and the number of smaller spines increased due to heterosynaptic LTD. There were also significant differences when comparing layers and time points. Sigma increased significantly ipsilaterally in the MML compared to the IML and OML at both 28 and 35 dpi, mirroring the skewness findings. The induction of plasticity appeared to broaden the spine size distribution, thus increasing sigma, by increasing the number of medium and large spines, which in turn also decreased skewness. Sigma also increased significantly over time in both the MML and OML, again mirroring the previous parameter comparisons.

**Figure 3.**
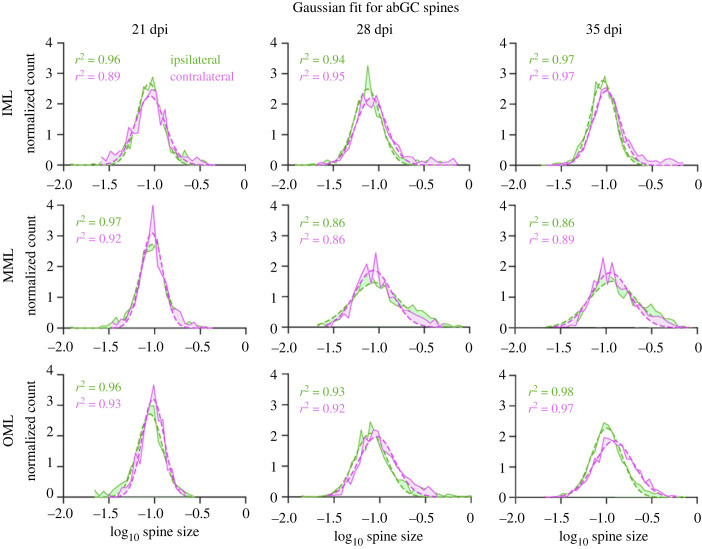
Fitting a Gaussian distribution to logarithmically transformed spine data of abGCs revealed that spine sizes follow a lognormal-like distribution. The average Gaussian fits for all cells in one dentate layer (OML, MML and IML), time (21, 28 and 35 dpi) and hemisphere (ipsi- and contralateral), fitted to the logarithm of the spine data (green and magenta). The dashed line shows the Gaussian fit, the solid line represents the spine data. The differences between data and fit are shown by shading in the areas between both. At 21 dpi, in all layers and both ipsi- and contralateral, the Gaussian distribution fits well to the data. In the older cells (28 and 35 dpi), there is apparent overabundance of bigger spines and thus a bias to the right of the peak. This is pronounced especially in the MML, where HFS occurred. OML, MML and IML: outer, middle and inner molecular layers, respectively; GCL: granule cell layer of the DG.

For a lognormal distribution, the logarithm of the individual values is normally distributed. As an additional quantification method, we calculated the logarithm of the data and fitted a Gaussian distribution to the transformed data (figure 3). The distributions at the youngest cell age (21 dpi) showed a well-fitted Gaussian distribution in all three layers and both ipsi- and contralaterally, indicating the condition for the lognormal distribution was met. In older cells (both 28 and 35 dpi), the Gaussian distribution fit less well to the logarithmic data. This was especially the case on the right side of the peak, where the actual number of spine sizes was higher than the estimated fit. There was an overabundance of bigger spines at older cell ages, regardless of plasticity induction. However, this overabundance of bigger spines could be observed especially in the MML, where homosynaptic plasticity was induced. This indicates that spines do not follow a strict lognormal distribution but a lognormal-like distribution.

We compared three skewed distributions, including the lognormal distribution, to quantify whether the lognormal distribution is the best fit of those three. To this end, we used the Akaike Information Criterion (AIC). Our analyses and comparisons revealed that of the three distributions tested (lognormal, gamma and Weibull), the lognormal distribution had an advantage over the other two, indicating that it was the best fit for the data (electronic supplementary material, figures S5 and S6).

### 2.2. Independence of spine size distribution from presynaptic transmitter release

Viewed together, the data from *in vivo* rat abGCs showed a strong independence of the lognormal-like spine size distribution from homosynaptic and heterosynaptic plasticity. This raises the question as to whether synaptic activity in general affects spine size distributions. To assess this, we analysed spines in nerve cells with blocked presynaptic transmitter release.

We used a dataset of CA1 pyramidal cell (CA1 PC) spines from organotypic hippocampal cultures obtained from Munc13-1/Munc13-2 DKOs [36]. In these mutants, presynaptic glutamate and GABA release is almost entirely blocked [36,37]. The spine data comprised three developmental time points, at which spine size was measured in organotypic slices (7, 14 and 21 days *in vitro*, div) and two further groups, one where synaptic activity (presynaptic transmitter release) was blocked (DKO group 0) and the corresponding control group (group 1). CA1 PCs possess three different spine types: 22.85 ± 6.01% mushroom spines (mean ± s.d.), 23.73 ± 4.83% thin spines and 51.16 ± 6.62% stubby spines. About 2.26 ± 2.53% were defined as ‘other’ and not included in further analyses.

The data were analysed by different conditions, separated by time *in vitro* (div) and group. In the first step, all cells and spine types were analysed together in each condition. In the second step, spine sizes were analysed at the single-cell level, for all spine types together. Finally, the three different spine types were analysed separately, first for all cells in one condition, then at the individual cell level as well.

**Figure 4.**
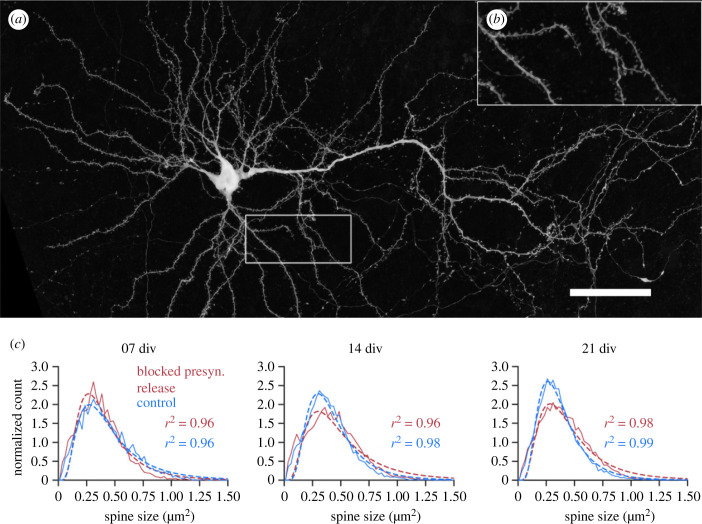
Collected spine size data from CA1 PCs in Munc13 DKO (blocked presynaptic release) and WT (control) organotypic slice cultures revealed robust lognormal-like distribution in all cell culture ages irrespective of blocked presynaptic release. (*a*) An example GFP labelled CA1 PC from a DKO slice culture imaged at 21 days *in vitro* (div; scale: 50 µm). (*b*) The panel shows an enlarged dendritic segment. (*c*) Average lognormal fit for all spines (mushroom, stubby and thin) and all CA1 PCs in one condition (blocked presynaptic release or control) pooled together. Note that the lognormal function fits the data (red and blue dots) with high goodness of fit (*r*^2^) values in both groups and at all time points (div). The dashed line shows the lognormal fit; the solid line represents the spine data.

A lognormal distribution was fitted to the spine data. As with the abGC data above, the goodness of fit (*r*^2^) showed that the lognormal fit described the spine size distribution very well, in all conditions and for all spines (figure 4).

**Figure 5.**
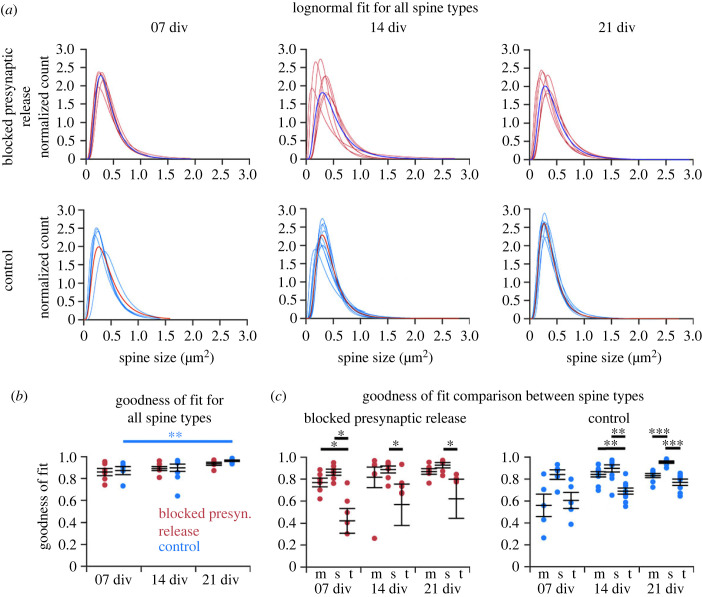
Individual cell level analysis of the spine size data from CA1 PCs in Munc13 DKO (blocked presynaptic release) and WT (control) organotypic slice cultures revealed a robust lognormal-like distribution independent of synaptic activity. (*a*) Lognormal fits in individual cells in both groups and at three time points (div). The single blue (above) and red (below) line represents the mean of all spine sizes as seen in figure 4*c*. (*b*) Goodness of fit (*r*^2^) comparisons. The comparison between the two groups yielded no significant difference. In the control group, *r*^2^ increases significantly (*p* < 0.01) over time. (*c*) Thin spines showed lower goodness of fit than stubby and mushroom spines in both experimental conditions. The *y*-axes are cropped at 0, with outliers below this value for thin spines in the blocked presynaptic transmitter release condition at 7, 14 and 21 div. Each point represents a single cell: blocked presynaptic release group: *n* = 6 cells (7 div), *n* = 7 (14 div), *n* = 6 (21 div); control group: *n* = 5 (7 div), *n* = 9 (14 div), *n* = 8 (21 div); error bars represent s.e.m. with mean. m: mushroom spines; s: stubby spines; t: thin spines.

Again, like with the abGC data, at the individual cell level, spine sizes in every CA1 PC in both groups followed a lognormal distribution, at each cell culture age (div) that we studied (figure 5*a*). There were differences in the shape and width of the distribution, but the rightward skewness was preserved even at the individual cell level.

We compared the cell individual goodness of fit parameter *r*^2^ between the groups and different time points (div), for all spines together (figure 5*b*). There were no significant differences between the two groups, only a trend in the blocked activity group towards a slightly reduced *r*^2^. Comparing the time points, there was no significant difference (*p* < 0.05) within the blocked activity group. In the control group there was a significant increase (*p* < 0.01) in the goodness of fit from day 7 to day 21 *in vitro*, indicating that the lognormal distribution described the data better for more mature slice cultures. A similar trend was seen in the blocked activity group, but without reaching statistical significance. This shows that there is a lognormal-like distribution of spine sizes irrespective of whether the presynaptic transmitter release is blocked or not.

A closer analysis of the spine size data revealed that the skewness values were typically above 0 (in some exceptional cases for thin spines below 0, indicating a skewness to the left), confirming that the spine sizes were not symmetrically distributed. Comparing the different conditions revealed no significant differences between different time points (cell culture age in div) or between the groups (electronic supplementary material, figure S7A). Within each group, the skewness increased slightly but not significantly over time. The sigma comparison revealed no significant differences in the width of the distribution and the range of spine sizes (electronic supplementary material, figure S8). A trend was seen at 21 div, where the blocked presynaptic transmitter release group has a slightly increased sigma compared to the control group, indicating that the range of spine sizes increases.

**Figure 6.**
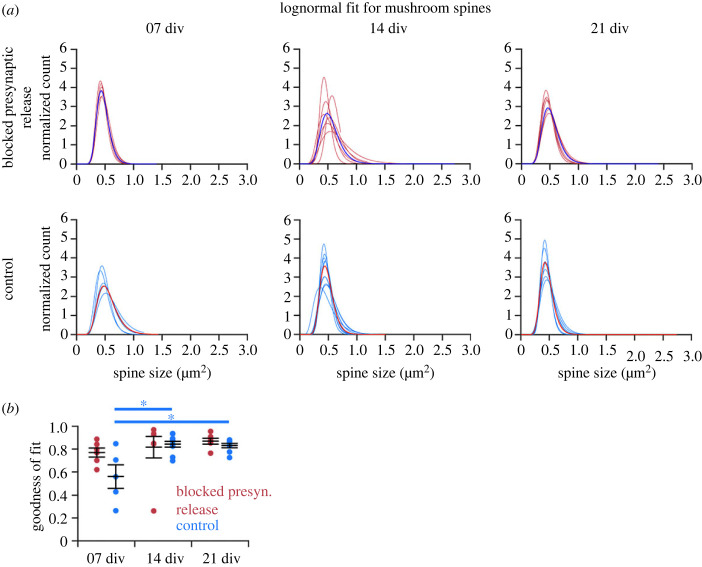
Analysis of mushroom spines from CA1 PCs in Munc13 DKO and WT (control) organotypic slice cultures showed a high goodness of fit to a lognormal distribution. (*a*) Individual fits for mushroom spines in each cell. The single blue (above) and red (below) fit shows the average distribution. (*b*) Goodness of fit (left panel) analysis revealed no significant differences between the groups (blocked presynaptic release versus control) and a significant increase in *r*^2^ over time (*p* < 0.05) (7, 14 and 21 div) for control. Each dot represents a single cell; error bar represents s.e.m. with mean.

Next, we tested whether a deeper analysis of spine type subgroups (mushroom, stubby and thin) would show inter- or intra-group differences (figure 5*c*). The thin spine population showed lower *r*^2^ values than the mushroom and stubby spine population. In line with this, thin spines also showed the lowest score for skewness (electronic supplementary material, figure S5B). At the individual cell level, mushroom spines in each cell followed a lognormal distribution (figure 6*a*). The group with blocked presynaptic transmitter release showed a similar goodness of fit as the control group. There was a significant increase of *r*^2^ over time (*p* < 0.05) in the control group (figure 6*b*). Mushroom spines had a slightly higher skewness in the control group, but the difference was not significant (electronic supplementary material, figure S7C), and they showed the lowest sigma value in comparison to the other two spine types, indicating a smaller range of sizes (electronic supplementary material, figure S8). Analyses of thin and stubby spines at the individual cell level are shown in electronic supplementary material, figures S9 and S10.

As with the abGC dataset, we conducted the AIC analysis and comparison for mushroom spines, to check whether the lognormal distribution was the best fit out of three skewed distributions. The lognormal distribution had an advantage over the other two in both experimental groups and at all cell ages (electronic supplementary material, figure S11). These findings indicate that a lognormal-like spine size distribution is preserved even when synaptic activity is blocked. Intriguingly, the sizes of thin spines showed a less good fit to a lognormal distribution.

**Figure 7.**
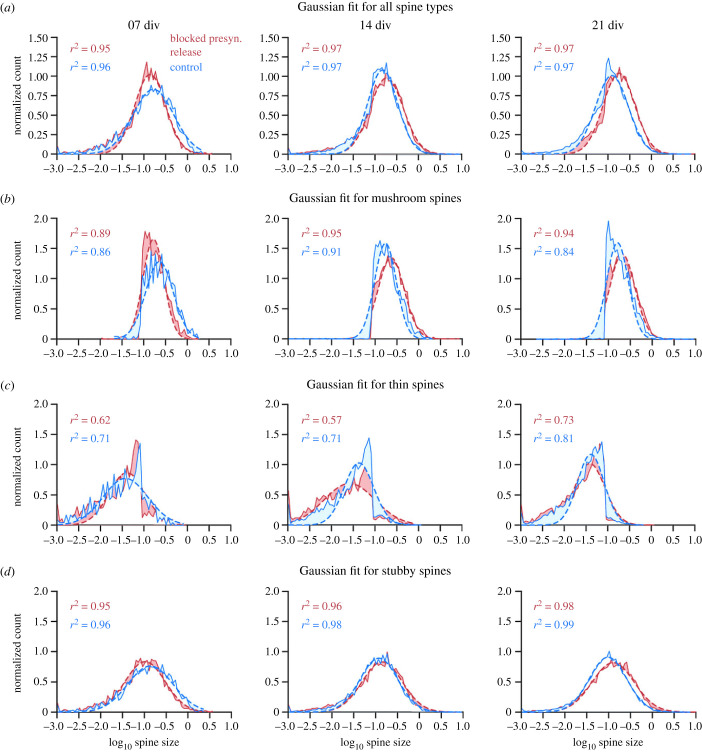
Gaussian fits to logarithmically transformed spine data for all spine subtypes and each type individually showed varying degrees of lognormality. The average Gaussian fits for all spine subtypes (*a*), mushroom spines (*b*), thin spines (*c*) and stubby spines (*d*) in all cells at one time (7, 14 and 21 div) and experimental condition (blocked presynaptic release and control), fitted to the logarithm of the spine data (red and blue). For all spine types together, there was a bias to the left of the peak at all three cell ages and for both conditions, indicating an overabundance of small spines in the data sample, making the distribution more lognormal-like. Mushroom spines (*b*) and thin spines (*c*) showed a cutoff at the same spine size, with mushroom spines displaying spine sizes above the cutoff and thin spines below. The Gaussian distribution did not fit as well to those two spine types, meaning that they are more lognormal-like. Stubby spines (*d*) showed the best fit to the logarithmic data. The dashed line shows the Gaussian fit; the solid line represents the spine data. The differences between data and fit are shown by shading in the areas between both.

Again, as with the abGC spine dataset, a final analysis of spine data from Munc13 DKOs and control littermates focused on the lognormal-like distributions of spine sizes in more detail by employing the normal (Gaussian) fits of logarithmically transformed data. The logarithm of lognormal-like spine size data should lead to a normal-like distribution. Taking the logarithm of the data and fitting a Gaussian distribution to the transformed data revealed for all spine types that the distribution had a bias towards the left side of the peak, meaning there was an overabundance of small spines in the samples (figure 7*a*), at all cell ages and in both experimental groups. For mushroom spines, there was a clear cutoff to the left (figure 7*b*), whereas for thin spines, there was a cutoff to the right of the peak (figure 7*c*). This was due to the method by which spines were categorized by size into thin or mushroom spines. Stubby spines showed the best Gaussian fit, indicating that the stubby spines were distributed strictly lognormally. The bias to the left might be an artefact of the method used to detect and measure the spines. Overall, the findings indicate, similar to the abGC dataset, that spines were lognormal-like distributed independently of synaptic activity.

### 2.3. A computational model implementing intrinsic and extrinsic synaptic plasticity accounts for the generation and preservation of skewed synaptic weight distributions

Many computational models of synaptic dynamics presume that the distribution of synaptic weights arises predominantly due to activity-dependent (extrinsic) synaptic plasticity [38–41]. Therefore, our observation that synaptic activity is not necessary for the emergence of skewed spine size distributions provides an opportunity to assess the relative contribution of activity-independent (intrinsic) plasticity in a model of synaptic dynamics. We therefore used a computational model of synaptic dynamics that combines intrinsic plasticity [32] with classical extrinsic plasticity mechanisms.

**Figure 8.**
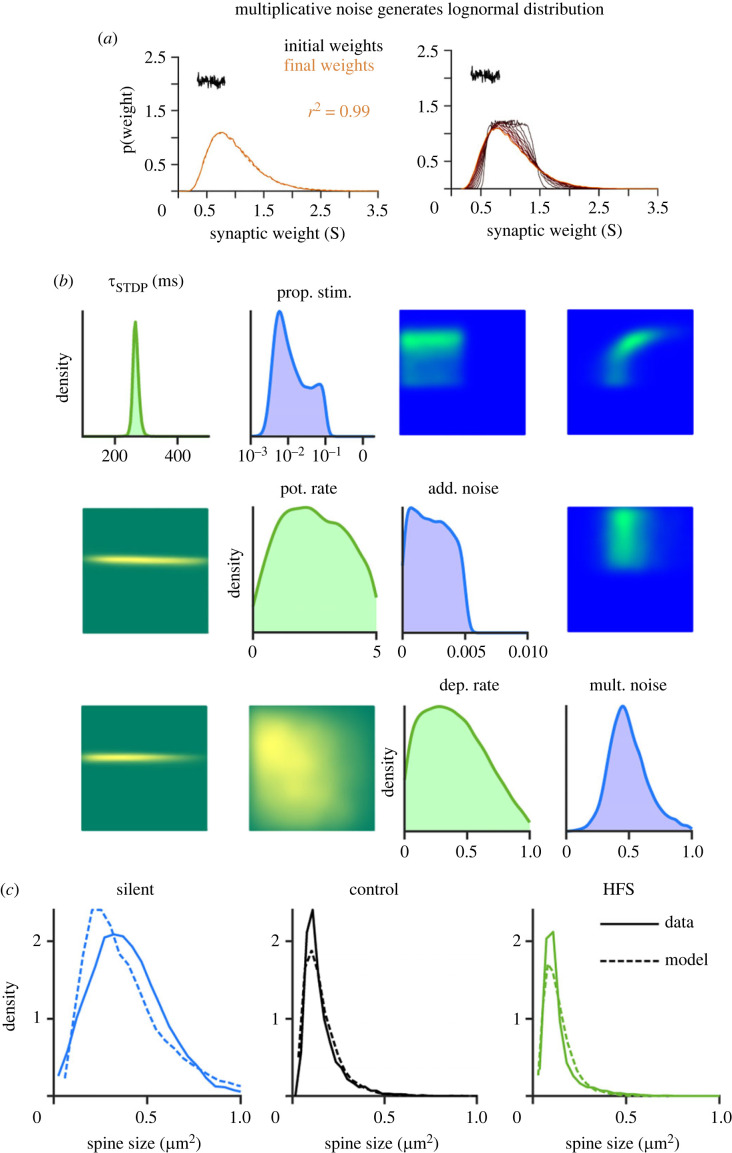
Computational modelling indicates that intrinsic (activity-independent) synaptic plasticity is sufficient to generate lognormal-like spine size distributions and a combination of intrinsic and extrinsic synaptic plasticity is sufficient to maintain lognormal-like distributions. (*a*) An initially uniform distribution becomes lognormal over time. Only multiplicative noise, representing intrinsic synaptic plasticity was applied to the distribution, which was sufficient to transform the uniform distribution into a lognormal distribution over time. (*b*) Single and pairwise marginals of model parameters that are consistent with the experimental data in the silent, control (10 Hz) and HFS (200 Hz) experimental cases. Intrinsic plasticity parameters are shown in the top right (blue) and extrinsic plasticity parameters are shown in the bottom left (green). All distributions are normalized. The parameters are the proportion of spines that receive stimulation, the proportional strength of multiplicative noise per second, the absolute strength of additive noise per second, the timescale of STDP (in ms), the absolute rate of potentiation and the relative rate of depression. (*c*) Observed (solid) and maximum *a posteriori* (dashed) distributions of spine sizes under different stimulation conditions.

Lognormal distributions are typically preserved when applying multiplicative stochastic operations. Combined intrinsic and extrinsic synaptic plasticity might represent a biological implementation of such multiplicative changes of synaptic weights. Thus, to investigate the influence of intrinsic and extrinsic plasticity on the lognormal distribution of spine sizes, we developed a minimal computational model, that was able to account for the experimental data. Extrinsic synaptic plasticity was modelled as Hebbian activity-dependent spike-timing-dependent plasticity (STDP) consisting of additive LTP and multiplicative LTD. Intrinsic synaptic plasticity was based on activity-independent fluctuations modelled as a combination of additive [42] and multiplicative noise. The model was inspired by van Rossum *et al*. [43]. The synaptic weights, for which we assume spine sizes to be a reliable proxy, were determined for each condition after the simulation was run, and a lognormal distribution was fitted over the weight data. In a first simulation, we wanted to see if intrinsic plasticity alone (modelled as multiplicative noise) can generate a lognormal distribution. To this end, we fed a uniform distribution as initial weights into the model and tracked the synaptic weights over the time course of the simulation to see how it developed (figure 8*a*). The distribution became lognormal over time, showing that multiplicative noise is indeed sufficient to generate lognormal distributions [32].

Next, we explored *in silico* how different modelling parameters could contribute to the range of different lognormal-like distributions produced by different experimental protocols. To do this, we combined our model with simulation-based inference [44,45], which uses simulated outputs to infer the distributions of parameters that could have given rise to the observed experimental outputs. We used the model to reproduce the plasticity processes in the Jungenitz *et al*. [33]) dataset and infer the possible underlying parameters. We compared a high-frequency stimulation (HFS) (periodic spiking input at 200 Hz) with a control simulation (Poisson input at 10 Hz) and with silenced tissue. Figure 8*b* plots the marginal single and pairwise distributions of parameters associated with intrinsic (top right) and extrinsic (bottom left) synaptic plasticity. Interestingly, the parameters did not typically show strong pairwise dependencies. There were two exceptions to this: the proportion of spines receiving external stimulation was positively correlated with the strength of multiplicative noise and the strengths of potentiation and depression under STDP were negatively correlated. Running simulations with the maximum *a posteriori* parameters produced lognormal-like distributions of spine sizes that closely matched the experimental data (dashed versus solid lines in figure 8*c*).

As shown by Rossum *et al.* [43], additive STDP can contribute to a skewed distribution of synaptic weights as already strong synapses are more likely to trigger a postsynaptic response and therefore potentiate again. Interestingly, however, additive intrinsic noise can lead to relatively large changes in the strengths of small synapses and limit the skewness of the resulting weight distributions. We found that additive noise should be relatively weak (if at all present) in order to maintain lognormal-like distributions of spine sizes.

In sum, and in agreement with the abGC spine data, combined extrinsic and intrinsic plasticity can maintain the skewed distributions in the presence of correlated LTP-inducing synaptic activation. Furthermore, in line with Munc13 DKO spine size data, our modelling shows that extrinsic plasticity is not necessary for the generation of skewed spine size distributions and that intrinsic plasticity alone is sufficient.

## 3. Discussion

Excitatory postsynaptic potential sizes and spine head sizes have lognormal-like distributions [1–3,5–7,46,47]. Here, we confirm that spine size distributions follow a lognormal shape in both hippocampal dentate abGCs *in vivo* and in organotypically cultured CA1 PCs. In dentate abGCs, a lognormal-like distribution of spine sizes was present at all studied cell ages, irrespective of homo- or heterosynaptic long-term plasticity induction. Most strikingly, in CA1 PCs, spine size distributions were skewed and lognormal-like even in Munc13 DKOs, in which presynaptic transmitter release is entirely blocked. These data show that the lognormal-like distribution of spine sizes is activity and plasticity independent. The skewness of spine size distributions develops early in cell age without extrinsic influences related to presynaptic transmitter release and therefore seems to be determined intrinsically. However, we cannot exclude potential extrinsic influences that are not related to presynaptic transmitter release, such as trophic factors or adhesion proteins.

### 3.1. Independence of spine size distributions from extrinsic plasticity

Intriguingly, we detected robust lognormal-like distributions of spine sizes in young newborn GCs that had experienced homo- and heterosynaptic plasticity. This is in agreement with previous studies showing unchanged spine size, spine type distribution and spine numbers at 30 min and 2 h after homosynaptic LTP in dentate GCs and CA1 PCs, respectively [20,48]. Together with our previous work [33,49], these data indicate that high-frequency activation of synapses evokes their homo- and heterosynaptic plastic changes leading to a redistribution rather than an overall increase (or decrease) in spine size and synaptic strength. Consistent with this, we observed significant changes in the width of the distributions: induction of plasticity broadened the spine size distribution in the MML by increasing the number of medium and large spines. At 35 dpi, the onset of both homo- and heterosynaptic plasticity [33], a concurrent narrowing of the distribution in the surrounding OML and IML can be observed. This is likely due to heterosynaptic plastic changes in these layers that reduce the size of medium and large spines, further pointing towards a redistribution of synaptic resources. The plasticity-related redistribution of synaptic weights with a homeostatic maintenance of the total synaptic area per micrometer of dendrite length [20,50] may be a result of activity-dependent competitive redistribution of synaptic building resources [51]. In addition to the plasticity-independence, the skewed spine size distribution in abGCs was detected at the earliest studied time point (21 dpi), shortly after onset of spinogenesis between 16 and 18 dpi [34,35]. This indicates that it develops in early stages of a nerve cell's life. Extending long-term time lapse imaging of abGCs [35] to include their initial developmental stages with the time of rapid spinogenesis should clarify whether the first spines already display skewed size distributions.

A recent study on cultured primary cortical neurons [32] provided results in line with our observation that spine size distribution is independent of presynaptic glutamate release. In this study on dissociated neurons in culture with pharmacologically blocked spiking and synaptic activity during the plating procedure, synapses showed physiological diversity with a full range of synaptic sizes [32]. The synapse size distributions in these silenced networks in culture were rightward skewed, broad and stable, showing characteristics of a lognormal-like distribution. Interestingly, networks with chronic activity suppression showed an increase in average spine size, and synaptic size distributions broadened, indicating that activity-dependent processes constrain synaptic growth [31,52,53]. Our analysis of spines upon blockage of presynaptic transmitter release documents a similar shift in spine sizes. The blocked transmitter release group shows a broader distribution with a lower peak, indicating a shift towards an increased number of bigger spines, possibly regulated by intrinsic mechanisms. Similar results were reported by Yasumatsu *et al*. [54] who observed individual spines of CA1 PCs from rat hippocampal slices in culture after blocking synaptic transmission and plasticity mediated by NMDA receptors. They reported that spontaneous, intrinsic spine volume fluctuations were independent of activity-dependent plasticity processes. In the presence of NMDAR inhibition, the rate at which spines were eliminated was decreased and spine generation was unaffected. Spine elimination of mostly small spines was reduced but new, small spines still emerged, affecting the skewness of the distribution.

An important finding of Yasumatsu *et al*. [54] was that small spines were the most plastic ones, changing in size, being eliminated, or newly generated even within one day. Large spines, in contrast, were more persistent. This supports the idea that small, more plastic spines are more involved in learning processes, whereas stable, large spines are responsible for memory traces [50,55,56]. This might hint at a potential advantage of lognormal size distributions, with a large pool of small spines with higher plasticity potential and a minority of big and less plastic spines that can hold long-term memory traces [57]. However, our present study and previously published data [32,36,58,59] show clearly that synaptic activity is not necessary for the emergence of large spines [31]. In line with this, the diversity of spine types—in terms of fractions of mushroom, stubby and thin spines—is not affected in mice with a complete suppression of synaptic transmitter release from glutamatergic neurons upon Cre-inducible expression of tetanus toxin [58,60]. Consistently, spinogenesis in CA1 PCs has been shown to be independent of the activation of ionotropic glutamate receptors [61], although their numbers might be modulated by the lack of activity [32,36]. Even the complete knockout of Ca^2+^ channels in synapses in cultured hippocampal neurons did not impair synapse structure [62]. All these observations are congruent with early investigations showing that *in vivo*-like synapse diversity emerges in neurons in chronically silenced organotypic cultures [63–65] (but see [29]).

### 3.2. Computational model accounts for the generation and maintenance of lognormal-like weight distributions

The finding that synaptic activity is not necessary for the skewed spine size and synapse weight distribution is unexpected in the context of several prominent theoretical models. Many computational models of synaptic weight dynamics assume that realistic weight distributions emerge due to a combination of Hebbian and non-Hebbian activity-dependent synaptic plasticity. For example, spiking network simulations led to the suggestion that a highly skewed distribution of synaptic weights appears due to network self-organization [39], by the combined effects of (i) excitatory and (ii) inhibitory spike-timing-dependent plasticity (STDP and iSTDP), (iii) synaptic normalization (preserving the total input weight of a neuron), (iv) intrinsic plasticity of neuronal excitability (for firing rate homeostasis) and (v) structural plasticity (in the form of synaptogenesis).

Similarly, other computational studies [43] used an STDP rule with a homeostatic component (diminished potentiation for strengthened synapses; see also [40]) or log-STDP [38] to reproduce the experimentally observed positively skewed weight distribution. Further, a more recent mathematical study argued that Hebbian learning is needed to produce and maintain skewed synapse size distributions [41]. However, the studies including our work and work of others [32] clearly show that activity-dependent synaptic plasticity is not essential for the lognormal-like weight distributions to occur. This means that the synaptic plasticity rules proposed in these computational studies are not necessary for the generation of heavy-tailed synaptic weight distributions, but that they may still be involved in the maintenance of the skewed distributions once neuronal networks become exposed to prolonged synaptic activity and plasticity.

Indeed, our plasticity model, using a Kesten process as multiplicative noise for implementing intrinsic synaptic fluctuations [32], generated a lognormal-like distribution without any influence of an extrinsic plasticity mechanism. The multiplicative noise (i.e. intrinsic synaptic plasticity mechanisms) also generated a lognormal distribution that is slightly broader than a control simulation with noise and activity-dependent plasticity (i.e. both intrinsic and extrinsic mechanisms). This is in accordance with our results obtained with the Munc13 DKO dataset. When we added additive STDP and simulated the network model with periodic high-frequency input (mimicking LTP-inducing activity), the skewed, lognormal-like distribution was maintained, but changed in width and shape compared to a control simulation that received 10 Hz input. The maintenance of the lognormal-like distribution is in agreement with the abGC LTP/LTD dataset. The insight of this model, as previously shown by van Rossum [43], is that even additive potentiation can generate and preserve skewed synaptic weight distributions as stronger synapses are more likely to trigger postsynaptic spikes and therefore more likely to undergo potentiation. The presence of skewed distributions even without STDP in our data is evidence that intrinsic noise is likely to be multiplicative and this is confirmed by our inference over the modelling parameters.

Activity-independent computational models based on stochastic multiplicative shrinkage and additive growth of synapses (mathematically well approximated by stochastic Kesten or nonlinear Langevin processes) successfully account for the emergence of lognormal-like synaptic strength distributions [32] (see also [1,54]). Similarly, a mechanistic model based on activity-independent cooperative stochastic binding and unbinding of synaptic scaffold molecules can explain the rightward skewed, distributions of synaptic sizes [32,66]. Our new model of intrinsic and extrinsic plasticity shows how activity-independent and activity-dependent synaptic dynamics may cooperate to maintain lognormal-like distribution of synaptic efficacies.

An open question that remains is as to whether long-tailed distributions of synaptic weights have functional relevance. Their computational role is still not fully understood but several studies indicate that they may support optimal network dynamics in the form of sparse, fast, broad and stable responses [4,5,67–69] and facilitate network burst propagation [70]. Sparse and strong synapses connect together to a so-called ‘rich club’ of rare but highly connected neurons [71,72]. The rich club neuron organization can generate bistable low-firing and high-firing network states, whereas biologically unrealistic random networks only display mono-stable, low-firing states [73]. The rare and strong synaptic connections participate to a disproportionate degree in information processing [72], such as feature preference and selectivity in visual cortex [4]. They may also contribute to memory recall in associative memory networks [74]. Network simulations also indicated that lognormal-like synaptic distributions are important in the context of criticality since they support continuous transitions to chaos associated with the generation of scale-free avalanches [75]. In addition, a recent computational study showed that strong synaptic inputs from the heavy tail of the lognormal synaptic efficacy distribution play a crucial role in triggering local dendritic spikes [76] which are known to enhance nonlinear single-cell computations.

### 3.3. Conclusion

In sum, our work highlights the importance of a skewed, lognormal-like distribution of brain parameters. It persists through HFS and plasticity processes, and emerges even when presynaptic transmitter release is blocked. Given its importance and widespread presence in the brain, computational plasticity models should strive to maintain a skewed, lognormal-like distribution of spine sizes and synaptic weights.

## 4. Methods

### 4.1. Spine data from dentate adult-born granule cells in rats with induced homo- and heterosynaptic plasticity

We analysed the distribution of spines in GC data in the DG, from Jungenitz *et al*. [33]). In this dataset, structural homo- and heterosynaptic plasticity of spines was induced in abGCs using 2 h HFS of the MPP in anaesthetized rats. AbGCs were stimulated at different time points after the injection of retroviral vectors (days post-injection, or dpi). The cell ages used in the analysis were 21, 28 and 35 dpi. The HFS induced LTP associated with spine expansion in the MML of the DG [33]. Concurrently, it induced heterosynaptic LTD associated with spine shrinkage in the IML/OML.

The dataset comprised spine data for individual cells in (i) the three different layers (IML, MML and OML), (ii) at the three different cell ages (21, 28 and 35 dpi) and (iii) from both the contra- and ipsilateral hemisphere. The contralateral side without the induction of synaptic plasticity [33] was included as a control. All analysed spines were mushroom spines (spines with a large head in relation to the neck [77,78]). Analysis was done at the level of individual cells or dentate molecular layers, separately for each layer, hemisphere and cell age. There were 12 cells available for analysis at 21 dpi ipsilaterally and 9 cells for the contralateral side. At 28 days, both ipsi- and contralateral sides consisted of 18 cells, and at 35 dpi, the ipsilateral hemisphere consisted of 30 cells and the contralateral side included 24 cells.

### 4.2. Spine data from CA1 pyramidal cells in Munc13 double-knockouts

The blocked presynaptic activity dataset contained spine data from CA1 PCs in hippocampal organotypic slices from Munc13 DKO mice [36]. In these DKOs, the elimination of synaptic protein Munc13 causes a complete loss of spontaneous and evoked transmitter release [37]. The dataset comprised spine data from M13-DKOs and their controls, from three different time points of measurement (7, 14 and 21 days *in vitro*, div). The dataset was split into apical and basal dendrites, and in three spine subgroups (mushroom, stubby and thin).

### 4.3. Fitting a lognormal distribution to the data

The spine head area was used to analyse the distribution of spine sizes. All analyses were done with Matlab software using a custom-written script. We analysed cells individually as well as collectively by combining and averaging all cells for one condition.

From the raw data, the mean (*µ*) and standard deviation (*σ*) of the spine sizes' natural logarithms were calculated. They functioned as a starting point for the algorithm implemented to fit the lognormal distribution over the spine data. Because the data spans multiple scales, the raw size data was normalized. For the normalization, the integral of the spine size distribution was calculated, and the absolute number of spines in each size bin was divided by that integral.

The next step in the analysis was to build the lognormal function that would be fitted to the normalized data. For this, a customized fitting procedure had to be derived for which the probability density function of the lognormal distribution was used:

$$f(x)=\frac{1}{x}\frac{1}{\sigma \sqrt{2\pi }}\exp\left(-\frac{{\left(\ln(x)-\mu \right)}^{2}}{2\sigma^{2}}\right)$$

*µ* and *σ* are defined as parameters, *f*(*x*) as the dependent and *x* as the independent variable. The lognormal distribution was then fitted to the normalized data. With the fit function, plots and respective goodness of fit statistics for each of the fits were generated. The goodness-of-fit statistics give an indication of how well the respective fit or model fitted the data. The r-square (*r*^2^) value was used in all further analysis.

The key characteristic of a lognormal distribution is that the logarithm of the random variable will be normally distributed. Thus, taking the logarithm of the spine data is another good method to check if the data is distributed lognormally like. A similar fitting procedure as above was applied. The data was first transformed by taking the logarithm of the spine sizes, then a Gaussian distribution was fitted to the logarithmic data:

$$f(x)=a\exp{\left(-\left(\frac{x-b}{c}\right)\right)}^{2},$$

where *a*, *b* and *c* are the parameters, *f*(*x*) the dependent and *x* the independent variable. With the fit function fitting a Gaussian distribution to the logarithm of the data, new plots were generated that compared the logarithmic data with the fit.

To determine differences between the different layers, cell ages or experimental and control groups, the given *r*^2^ for each condition was compared, using statistical non-parametric tests. *r*^2^, or the coefficient of determination, is used to determine how well the variation in f(*x*) (the dependent variable) can be explained by *x* (the independent variable(s)). Essentially, it provides a measure of how well the observed outcomes can be replicated by a model. In our case, how well the applied fits describe the spine size data. The value is less than or equal to 1, with 1 being a perfect fit of the model. The coefficient of determination is calculated as follows:

$$r^{2}=\frac{\mathrm{explained}\;\mathrm{variation}}{\mathrm{total}\;\mathrm{variation}}.$$

To support the findings of the goodness of fit comparisons, we also looked at the skewness (asymmetry around the mean) of the data and the width of the distribution (standard deviation of the data's natural logarithm, in the following called sigma). More information about these comparisons can be found in the electronic supplementary material, methods. Additionally, we conducted a model fit comparison for which we fit two additional skewed distributions (gamma and Weibull) to the data and then used the AIC to compare all three distribution fits. This was done to see whether or not the lognormal distribution was the best fit for the data. More information about the AIC calculations and comparisons can be found in the electronic supplementary material, Methods.

### 4.4. Statistical analysis

Several statistical tests were applied to test for statistical differences of *r*^2^ for a lognormal and the skewness between the different conditions in both datasets. The distribution analysis showed a lognormal distribution in the spine data, so only non-parametric tests were applied.

For the hemisphere (ipsilateral / stimulated versus contralateral / non-stimulated) comparison in the rat dentate abGC spine data and the group comparison (Munc13 DKO group with blocked presynaptic release versus control group) in the mouse CA1 PC spine data, we used a Mann–Whitney *U*-test or rank-sum test. To compare between the three different dentate layers (IML/MML/OML), we used Friedman's test. Since all three layer-samples in one cell come from the same cell, it was a repeated measurement of multiple variables. The Kruskal–Wallis test was used for the comparison between different cell ages or cell culture ages. If significant differences (*p* < 0.05) were found in one sample, both for the time comparison and the layer comparison, *post hoc* paired rank sum tests were conducted. A Bonferroni-Holm correction for multiple tests was applied to test for specific significant differences in the sample.

### 4.5. Multiplicative spike-timing-dependent plasticity model to investigate lognormal distributions

To further investigate the influence of plasticity on the lognormal-like distribution of synaptic weights, we developed a simple model based on van Rossum *et al*. [43]. The model includes heterosynaptic scaling, intrinsic multiplicative (Kesten) and additive noise processes, and an STDP learning rule with additive potentiation and multiplicative depression. The times between a presynaptic event and a postsynaptic event are written as Δ*t*. Negative values of Δ*t*, where the presynaptic event precedes the postsynaptic event, lead to potentiation *w* → *w_p_* and positive values lead to depression *w* → *w_d_*.

$$w_{p}=w+c_{p}\exp\left(\frac{-\Delta t}{\tau STDP}\right)$$

and

$$w_{d}=w-wc_{d}\exp\left(\frac{\Delta t}{\tau STDP}\right),$$

*w* is the synaptic weight, *c_p_* is the weight of potentiation (*c_p_* = 0.007 pS), *c_d_* is the weight of depression (*c_d_* = 0.003) and τ is the time constant for STDP (τSTDP=0.5 ms). In addition, the synapses are affected by continuous-time multiplicative and additive Gaussian noise processes. The strength of multiplicative noise is given in proportion of spine size per second, and the strength of additive noise is given in absolute units per second. The postsynaptic neurons are modelled as leaky integrate-and-fire cells receiving 100 inputs each and uniform heterosynaptic scaling maintains a constant total conductance. Only a proportion of the inputs receive a given stimulation protocol. The membrane time constant is 10 ms and the firing threshold is 10 mV above rest.

The model consists of a population of 1000 neurons, and the synapses that receive inputs are stimulated either in a Poisson manner or with periodic spiking, at different input frequencies depending on the simulation condition.

To see whether or not multiplicative noise (i.e. intrinsic mechanisms) is enough to generate a lognormal distribution, a uniform distribution was fed into the model as an initial distribution and the synaptic weights were measured throughout the simulation. The model was then used to replicate the three experimental datasets. First, the HFS that induced LTP in the stimulated spines was recreated with the model, using periodic spiking as input at a 200 Hz frequency. This was compared with a control simulation that received 10 Hz Poisson input. Finally, a silent simulation without inputs was considered. A lognormal distribution was fitted to the synaptic weight data in the same way as previously described. Additionally, the logarithm was taken of the data and a Gaussian distribution was fitted to the transformed data.

### 4.6. Simulation-based inference of spine-size data

Simulation-based inference with sequential neural posterior estimation [44,79] was used to infer the modelling parameters that could have produced the experimentally observed spine-size distributions. The model was run with the three input protocols described above and the lognormal parameters mu and sigma were fitted to the resultant spine distributions. This gave a set of six summary features that the inference could be fitted to. To convert from simulated synaptic weights to measured spine sizes, the simulated results were normalized to have the same mean as the experimental data in each case. Simulations were carried out sequentially in five rounds of 3200 starting with a uniform prior. The posterior was sampled 10 000 times to produce the marginal distributions shown in figure 8.

## Data Availability

Code is available in the open GitHub repository: https://github.com/NinaRoessler/Lognormal-like-skewed-distribution-of-spine-sizes. Data are already published in [33] and [36]. Additional information is provided in the electronic supplementary material [80].
